# Should chronic hepatitis B mothers breastfeed? a meta analysis

**DOI:** 10.1186/1471-2458-11-502

**Published:** 2011-06-27

**Authors:** Yingjie Zheng, Yihan Lu, Qi Ye, Yugang Xia, Yueqin Zhou, Qingqing Yao, Shan Wei

**Affiliations:** 1 Department of Epidemiology, School of Public Health, Fudan University, Shanghai 200032, China; 2 The Key Laboratory on Public Health Safety, Ministry of Education, Fudan University, Shanghai 200032, China; 3 Library of Medical Centre, Fudan University, Shanghai 200032, China

## Abstract

**Background:**

Hepatitis B virus (HBV) exists in the breast milk of chronic hepatitis B (CHB) mothers. The authors use a meta-analytic technique to quantify the evidence of an association between breastfeeding and risk of CHB infection among the infants vaccinated against HBV.

**Methods:**

Literature search is performed up to 2010 on the relationship between infantile CHB infection within one-year follow up after immunization with the third-dose hepatitis B vaccine and breastfeeding. Two reviewers independently extract the data and evaluate the methodological quality. A random-effects model is employed to systematically combine the results of all included studies.

**Results:**

Based on data from 32 studies, 4.32% (244/5650) of infants born of CHB mothers develop CHB infection. The difference in risk of the infection between breastfed and formula-fed infants (RD) is -0.8%, (95% confidence interval [CI]: -1.6%, 0.1%). Analysis of the data from 16 of the studies finds that RD for mothers who are positive for the HBeAg and/or the HBV DNA, 0.7% (95%CI: -2.0%, 3.5%), is similar to that for those who are negative for these infectivity markers, -0.5% (95%CI: -1.7%, 0.6%).

**Conclusions:**

Breast milk is infectious; yet, breastfeeding, even by mothers with high infectivity, is not associated with demonstrable risk of infantile CHB infection, provided that the infants have been vaccinated against HBV at birth.

## Background

The hepatitis B virus (HBV) accounts for a significant morbidity and mortality worldwide. An estimated one-third of the world population has been exposed to HBV and 400 million people are chronic carriers [1]. Mother to infant transmission of HBV would occur among up to 90% of infants born of chronic hepatitis B (CHB) mothers who are positive for the infectivity markers, the HBeAg and/or the HBV DNA [2]. With the introduction of a safe and effective hepatitis B vaccine, infantile CHB infection has dramatically dropped to around 5% [3].

The advantages of breastfeeding over formula feeding have been well documented [4]. However, the presence of the HBV in breast milk firstly reported by Linnemann [5] in 1974 and confirmed by later studies [6,7] raised the possibility of transmission of HBV through breastfeeding. But later studies found no association between breastfeeding and infantile CHB infection, both before and after the introduction of hepatitis B vaccine [8-13]. In the absence of evidence that breastfeeding poses any additional risk of infection to infants born of CHB mothers, World Health Organization (WHO) recommends breastfeeding even for area where HBV infection is highly endemic and HBV vaccine is not available [14].

Scepticism about this recommendation remains among clinicians, however. About 25% of obstetricians in Australia [15] and 50% of physicians (most of them were hepatologists!) in Illinois [16] did not recommend CHB mothers to breastfeed their babies. It was noted that 5.4% of pregnant women in Shanghai were HBsAg positive and 30.3% of whom were HBeAg-positive (Tao, personal communication), which was reported to pose a significant risk of HBV transmission to infants, including those who had been vaccinated against the virus [17]. In the light of this and other studies [10], it is a common and recommended practice that CHB mothers with an abnormal alanine aminotransferase (ALT) level before delivery do not breastfeed.

In the present paper, we conduct a meta analysis to assess the risk of CHB infection of vaccinated infants through breastfeeding associating with CHB mothers who are and are not positive for the HBV infectivity markers.

## Methods

### Data sources and search strategy

Two independent investigators (Ye and Zhou) searched PubMed (1966-), Chinese BioMedical Literature database (CBM, 1978-), Chinese National Knowledge Infrastructure (CNKI, 1979-) and Wanfang Data (Wanfang, 1998-) up to December 31^th^, 2010 by using the combination of MeSH terms "breast feeding" or "breastfeeding" and "hepatitis B". In addition, the reference lists of potentially relevant manuscripts were scanned backward to obtain extra eligible studies.

### Criteria for inclusion of the studies

(1) Follow-up studies, prospective or retrospective, on the association between breastfeeding (BF) versus formula-feeding (FF) and risk of CHB infection among the infants born of CHB mothers.

(2) All infants must receive at least 3 doses of hepatitis B vaccines, with or without receiving hepatitis B immunoglobulin (HBIG) after birth.

(3) CHB infection is defined as the presence of any of the three HBV markers (HBsAg, HBeAg and HBV DNA) in blood, during prenatal care visit and/or before delivery of their babies for the mothers and within one year after immunization with the third-dose hepatitis B vaccine for the infants.

### Data extraction

Two independent investigators (Xia and Yao) were involved in data extraction. The third investigator (Lu) examined the results, and a consensus was reached. The outcome, CHB infection of the infants born of CHB mothers at the end of follow-up within one year after immunization with the third-dose hepatitis B vaccine was considered. We extracted the following data from the eligible studies: authors' names, journal and year of publication, country of origin, enrolment periods, type of study, number of CHB and non-CHB among the infants by feeding methods, general characteristics of the babies and their mothers.

### Assessment of methodological quality

Two independent investigators (Yao and Wei) evaluated the quality of each study based on Newcastle-Ottawa Quality Assessment Scale (NOS) [18]. The third investigator (Zheng) examined the results, and a consensus was reached (Table 1). We did not consider the 4^th ^item under selection (Demonstration that outcome of interest was not present at start of study) and the 2^nd ^item under outcome (Was follow-up long enough for outcomes to occur) in NOS, for the reason that the outcomes were available at the end of the follow-up period which was pre-defined in our criteria of inclusion. And we defined the studies with NOS ≥ 5 as with high quality and the others with NOS < 5 as with low quality.

**Table 1:** General characteristic and quality score of the 32 studies included in this meta analysis

No	Author, publication year and NOS	Mothers enrolled period and sites	Number of infants, length of follow up and types of study	Characteristic of the mothers*^1^	Mothers' Infectivity%	Characteristic of the babies	Infantile vaccination*^2^	DBF (m)	Other characteristics
1.	De Martino[11] 1985 3	NA Italy	85 cases 12 months Prospective	APCW, S+ and C+; a normal pregnancy; informed consent.	2.3(E)	Normal ALT and birth weight.	0.5 ml/kg HBIG/8 h + 1 ml(P)/2-4-11 m		Sex ratio of 1.2, no medication except a multivitamin mixture, vaccination against polio, tetanus and diphtheria; monthly visit, no dropout; no vaccine-related serious side effect and no abnormal ALT was observed at the end of follow up. Mothers' E+% and newborns' gender were similar between BF and FF.
2.	Tseng [12] 1988 3	NA Hongkong, China	170 cases 12 months Retrospective	PCW, S+.	64(E) among BF		0.5 ml HBIG/24 h + 5 μg (P)/0-1-6 m		
3.	Huang [22] 1993 3	Aug. 1989-Sept. 1990 Guangdong, China	112 cases 12 months Retrospective	PCW, S+.	44.8(E)	S+% = 6.4 and E+% = 25.6 (U) at birth.	30 μg(P)/0-1-6 m	9-12	Average age of the mothers was 26.97 ± 2.83 years old.
4.	Wu [23] 1996 3	1992-1993 Nanjing, China	80 cases 6 months Retrospective	PCW, S+.	55.6(E) among BF	Full-term normal delivery.	200IU HBIG/4 h,1 m +30 μg(P)/1-3-6 m.	12	Mothers' age were similar between BF and FF.
5.	Zeng [24] 2001 4	Jan. 1996-Jun. 1999 Guangdong, China	190 cases 9 months Retrospective	PCW, S+; normal ALT.	29.5(E)	S+% = 14.9(U, 134cases) at birth.	10 μg (R)/0-1-6 m		Mothers' E+% was higher in FF than BF.
6.	Wu [25] 2002 3	Jan. 1995-Dec. 2000 Nanjing, China	300 cases 12 months Retrospective	PCW, S+; normal hepatic function; no nipple crack; 200IU-HBIG treatment.	100 (E)	Complete gestation birth; no oral ulcer.	200IU HBIG/4 h,1 m+10 μg (R)/1-3-6 m with booster.	11	Mothers' age range was 25-31 years.
7.	Hill [10] 2002 6	Jan. 1992-Sept. 1998 Texas, The United States	369 cases 9-15 months Prospective	PCW, S+, CG+ and CM-	24.5(E, 179 cases)		0.5 ml HBIG+ 0-1-6 mwith booster.	4.9	Complete follow-up and vaccination; mothers' nulliparous% (all cases), AST/ALT level, anti-HCV+%, HIV-1+%, E+%, CG+% and CM+% based on available cases, and newborns' gender were similar between BF and FF. Mothers were younger and with more blacks in FF than BF.
8.	Wang [13] 2003 5	1994-1999 Shanghai, China	230 cases 12 months Retrospective	APCW, S+; normal ALT.	34(E)	37-42 gestation weeks; S+% = 10.9 at birth.	30 μg(P, before 1997) or 5 μg(R, after 1997)/0-1-6 m or/24 h+1-2-7 m	≥ 2	Those infants were excluded with an obvious abnormality, birth weight < 2500 g, or an apgar score of < 8 at 1 or 5 minutes. Mothers' E+%, newborns' gender, birth weight and S+% at birth were similar between BF and FF. BF group received more active immunization than FF.
9.	Zeng [26] 2003 2	May 1995-Sept. 1997 Sichuang, China	53 cases 12 months Retrospective	PCW, S+.	100(E or DNA)	S-at birth.	200IU HBIG/24 h+30 μg(P)/2 w,10 μg/1.5 m, 10 μg/6.5 m	≥ 4	Duration of breastfeeding < 4 months was excluded.
10.	Gu [27] 2003 3	1998-1999 Nanjing, China	152 cases 12 months Prospective	PCW, S+ and c+; 200~400IU-HBIG treatment	0(E)	Healthy; complete gestation weeks; birth weight > 3000 g; S+% = 7.6 at birth.	10 μg(R, 80 cases) or 30 μg (P, 72 cases)/24 h-1-6 m		Proportion of types of vaccines was similar between BF and FF.
11.	He [28] 2004 3	Dec. 1997-Dec. 1999 Inner Mongolia, China	38 cases 12 months Retrospective	PCW, S+.	100(DNA)	DNA+% = 56(U)	100-200IU HBIG/6 h, 2 w+ 0-1-6 m		
12.	Liu [29] 2004 3	Feb. 1998-Feb. 2001 Henan, China	436 cases 12 months Retrospective	APCW, S+; 200IU-HBIG treatment.		S+% = 8.7 and E+% = 0.9 at birth	200IU HBIG/24 h, 2 w+5 μg(R)/0-1-6 m		Newborns' S+% and E+% at birth were similar between BF and FF.
13.	Yao [30] 2004 4	Dec. 2000-Jun. 2002 Zhejiang, China	190 cases 12 months Retrospective	PCW, S+; 200IU-HBIG treatment.	72.9(E)	Healthy; complete gestation weeks; DNA+% = 5.0 and S+% = 6.3 at birth.	200IU HBIG/6-12 h,2 w+5 μg(R)/0-1-6 m		Mothers' age, uterine contraction+%, duration of uterine contraction, E+% and HBV+%, and newborns' gestation weeks and birth weight were similar between BF and FF.
14.	Meng [31] 2004 4	Mar. 2001-Mar. 2003 Guangdong, China	134 cases 6 months Prospective	PCW, S+; normal ALT; 200IU-HBIG treatment (66 cases).		S+% = 16.4, E+% = 6.7 and DNA+% = 2.2 at birth.	0-1-6 m		No foetus abnormality, no history of treatment of anti-virus drugs during pregnancy, TORCH syndrome and other diseases.
15.	Mou [32] 2005 5	Sept. 2001-Oct. 2003 Shandong, China	91 cases 12 months Prospective	PCW, S+; normal hepatic function; informed consent.	33.0(E) 37.4(DNA)	DNA+% = 13.2 (U) at birth.	200IU HBIG/12 h,2 w+0-1-6 m with booster.	≥ 1	Exclusion for those with premature birth, low birth weight, asphyxia and history of bleeding during pregnancy, breastfeed < 1 month. Mothers' E+% and DNA+%, newborns' gestation weeks, birth weight and DNA+% (U) were similar between BF and FF.
16.	Zhang [33] 2005 4	2000-2004 Ningxia, China	104 cases 12 months Prospective	PCW, S+; 200IU-HBIG treatment.	100(E)		200IU HBIG/24 h, 1 m+10 μg(R)/2-3-6 m		Natural delivery% = 50.0
17.	Zhang [34] 2005 3	Jan. 2001-Feb. 2002 Chongqing, China	67 cases 12-18 months Retrospective	PCW, S+; 100IU-HBIG treatment.	28.4(E)		200IU HBIG/24 h+100IU HBIG/15d+10 μg(R)/2-3-6 m		Mothers' E+% was higher in FF than BF.
18.	Qiu [35] 2005 3	Dec. 2002-Mar. 2004 Zhejiang, China	90 cases 6 months Retrospective	PCW, S+; normal hepatic function; no nipple crack; 200IU-HBIG treatment.	100(E)	Healthy; complete gestation weeks.	200IU HBIG/6 h+5 μg (R)/12 h-1-6 m	> 6	Mothers' age and DNA+%, newborns' gestation weeks were similar between BF and FF.
19.	Zeng [36] 2006 4	Jun. 2001-Dec. 2003 Guangxi, China	115 cases 7 months Prospective	PCW, S+; normal ALT; 200IU-HBIG treatment.	31.3(E)	Apgar > 7; birth weight > 2500 g; S+% = 12.2 at birth.	200IU HBIG/24 h, 1 m+ 5 μg (R)/72 h-1-6 m	> 0.5	Mothers' E+%, newborns' gender, birth weight and HBV+% at birth were similar between BF and FF.
20.	Ma [7] 2006 4	1993-1999 Liaoning, China	167 cases 9 months Retrospective	PCW, S+.	54(E)	Birth temperature < 37°C; birth weight > 2500 g; apgar > 8.	(R)/0-1-6 m		Mothers' E+% was similar between BF and FF.
21.	Chen [37] 2006 4	Mar. 2002-May. 2005 Zhejiang, China	170 cases 12 months Retrospective	APCW, S+; normal hepatic function; informed consent.	41.2(E)	DNA+% = 9.4(U).	200IU HBIG/12 h,14 d+5 μg(R)/0-1 -6 m with booster	≥ 1	Exclusion for those with premature birth, low birth weight, asphyxia and history of bleeding during pregnancy, breastfeeding below one month. Mothers' E+% and DNA+%, newborns' birth weight, gestation weeks and DNA+% were similar between BF and FF.
22.	Zuo [38] 2007 4	May 2004-May 2005 Hebei, China	180 cases 12 months prospective	PCW, S+; 200IU-HBIG treatment.	49.4(E)	S+% = 6.7 and DNA+% = 4.2 at birth.	200IU HBIG/48 h,1 m,3 m+5 μg(R)/24 h-1 -6 m	10	The infants were randomized into two groups. Mothers' age, E+% and DNA+%, newborns' birth weight, method of delivery, S+% and DNA+% were similar between BF and FF.
23.	Wang [39] 2007 3	Oct. 2003-Jan. 2006 Ningxia, China	165 cases 10-12 months Retrospective	APCW, S+.	0(E)	S+% = 11.5 and E+% = 2.4.	200IU HBIG/6 h+5 μg(R)/2 d-1-6 m		Mothers' age and DNA level, newborns' sex ratio, birth weight and method of delivery were similar between BF and FF.
24.	Qin [40] 2007 3	Jan. 2001-Jan. 2005 Henan, China	74 cases 12 months Retrospective	PCW, DNA+; 200IU-HBIG treatment.	100(DNA)	DNA+% = 4.5 at birth	200IU HBIG/16 h+10 μg(R)/1-2-7 m or 10 μg(R)/0-1-6 m		
25.	Chen [41] 2008 4	Oct. 2002-Aug. 2007 Beijing, China	345 cases 12 months Retrospective	PCW, S+.	0(E and DNA)		10 μg(R)/0-1-6 m+ 100-200IU HBIG/24 h, 15-30d(241 cases).		Proportion of infants who received HBIG was higher in FF than BF.
26.	Hou [42] 2009 4	Jan. 2002-NA Shenzhen, China	369 cases 9-15 months Retrospective	PCW, S+; abnormal ALT/AST% = 2.2.	13.6(E)		100IU HBIG/1 d+5 μg(R)/0-1-6 m with booster.	4.9	Mothers' age, abnormal ALT/AST%, E+%, CM+% and CG+%, newborns' sex ratio were similar between BF and FF.
27.	Li [43] 2009 2	2004-2007 Shandong, China	45 cases 12 months Retrospective	APCW, S+; normal hepatic function; 200IU-HBIG treatment.	33.3(E)	DNA+% = 10.0 at birth.	200 IU HBIG/12 h, 2 w+ 0-1-6 m with booster.		Mothers' E+% and DNA+%, newborns' gestation weeks, birth weight and DNA+% were similar between BF and FF.
28.	Luo [44] 2010 4	2007-2009 Fujian, China	436 cases 6 months retrospective	PCW, S+; normal hepatic function; 200IU-HBIG treatment.	44.0(E)		200 IU HBIG/12 h, 2 w+5 μg(R)/0-1-6 m	> 6	Mothers' E+% and DNA+% were higher in FF than BF.
29.	Chen [45] 2010 3	2001-2009 Shandong, China	278 cases 8-12 months Retrospective	PCW, S+; 200IU-HBIG voluntary treatment.			200IU HBIG/24 h,2 w or 200IU HBIG/24 h +0-1-6 m or 0-1-2-7 m		Mothers' age range was 21-40 years old.
30.	Sun [46] 2010 2	Nov. 2007-Jul. 2008 Shandong, China	69 cases 12 months retrospective	PCW, S+.		DNA-at birth	HBIG+(R)/0-1-6 m		
31.	Liu [47] 2010 4	Jan. 2009-Mar. 2010 Guangdong, China	145 cases 12 months retrospective	PCW, S+; 200IU-HBIG treatment.	58.1(DNA)		200IU HBIG+5 μg (R)/0-1-6 m	> 6	Mothers' DNA+%, age, time of pregnancy and delivery, methods of delivery, newborns' birth weight, S+% and DNA+% at birth were similar between BF and FF.
32.	Wu [48] 2010 3	Sept. 2007-May. 2009 Guangdong, China	46 cases 12 months Retrospective	PCW, S+.	100 (both E and DNA)		200IU HBIG/6 h+5 μg(R)/0-1-6 m		Mothers' age, newborns' sex ratio, birth weight and methods of delivery were similar between BF and FF.

NOS: Newcastle-Ottawa Quality Assessment Scale; NA: not available; BF: breastfeeding; FF: formula feeding; hepatitis B immunoglobulin: HBIG; APCW: asymptomatic prenatal care women; PCW: prenatal care women; S: HBsAg; E: HBeAg; C: HBcAb; CG: HBcAb IgG; CM: HBcAb IgM; DNA: HBV DNA; P: plasma-derived vaccine; R: recombinant vaccine; U: umbilical cord blood; ALT: alanine aminotransferase; AST: aspartate aminotransferase; IU: international unit; m: month; w: week; d: day; h: hours; ml: millilitre; kg: kilogram; μg: microgram; DBF: duration of breastfeeding

*^1^: HBIG treatment: HBIG was received by the mothers at the gestation weeks of 28 th, 32 th and 36 th each.

*^2^: One boost dose was given to those infants with negative HBsAb status.

### Statistical analysis

The risk difference (RD) with 95% confidence interval (CI) was used as a measure of effect between breastfeeding (BF) versus formula-feeding (FF) and risk of infantile CHB infection across studies. The Dersimonian and Laird random-effects model was used to pool the RDs across studies in Stata version 10.0 (Stata Corp). Heterogeneity was explored by χ^2 ^test, with a significance set at a *P *value < 0.10. The extent of heterogeneity was measured by Higgins' I^2^. Subgroup analysis and Meta-regression were carried out to examine the effect in relation to quality and type of study, language of paper, study sites, hepatitis B vaccination of the mothers and infants, and mothers' infectivity if available. We used the test for interaction to estimate the difference between two subgroups [19]. Publication bias was assessed by visual inspection of a funnel plot, the Egger's and Begger's test [20], and a nonparametric trim and fill method was performed [21].

## Results

### 1. Literature search

The search retrieved 517 papers, and 99 out of which were potentially relevant to current analysis. Further backward search produced 3 additional papers. Only 32 papers [7,10-13,22-48] which offered 32 independent studies were included in our analysis and the reasons for exclusion of the others were listed in Additional file 1-Figure S1.

### 2. General characteristics about the studies included in meta analysis

The 32 studies included in present analysis were published between 1985 and 2010 (Table 1). One study was conducted in the United States [10], one in Italy [11] and the other 30 in China, including Hongkong. Mothers decided the feeding methods in all studies except one in which they were randomized [38]. The sample size of the studies ranged from 38 to 436 (median, 143), with the ratio of BF versus FF infants of 0.09-5.07 (median, 0.94).

### 3. The risk difference (RD) of infantile CHB infection with methods of feeding

**Figure 1:**
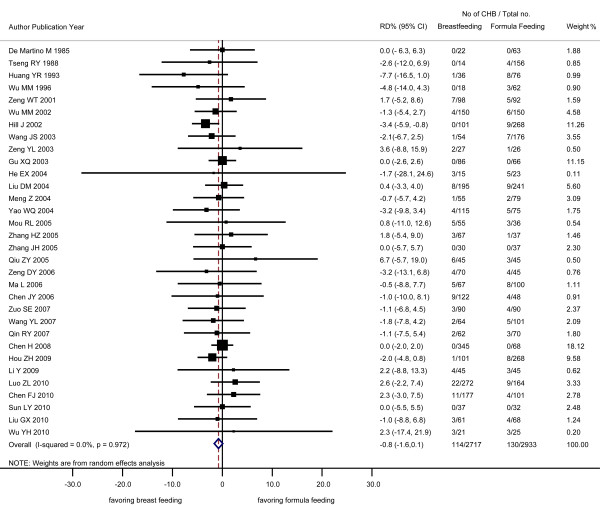
**Effect of breastfeeding on occurrence of chronic hepatitis B (CHB) infection among the infants born of CHB mothers analyzed by a random-effects model**. RD: risk difference; CI: confidence interval; CHB: chronic hepatitis B.

The 32 studies contributed 5650 infants and 244 CHB outcomes with an overall transmission rate of 4.32%. The difference between the risk of infantile CHB infection among BF and FF infants (RD) determined by the random-effects model was -0.8%, (95% confidence interval [CI]: -1.6%, 0.1%) (Figure 1) and that determined by the fixed-effects model was -0.4% (95% CI: -1.6%, 0.7%). The findings suggest that BF is not associated with additional risk of infantile CHB infection concurred with that of all the individual studies, except one [11], which suggests that BF is associated with a lower risk than FF.

Subgroup studies showed that the RDs could be modified by language of paper and quality of study, but not by type of study, study sites or infant-mother vaccination. The studies in English or with high quality tended to report lower RDs, which would probably reflect the difference of mothers' HBV infectivity (Table 2).

**Table 2:** Proportion of the mothers with high infectivity overall, by language and quality of papers

Groups	Breastfeeding		Formula feeding	
		
	No of high infectivity/ Total No	Proportion of high infectivity (%)	No of high infectivity/ Total No	Proportion of high infectivity (%)
All studies	739/2001	36.9	812/1843	44.1
Language of papers				
English	29/90	32. 2	60/239	25.1
Chinese	710/1911	37.2	752/1604	46.9
Quality of papers				
high	37/109	33.9	71/212	33.5
low	702/1892	37.1	741/1631	45.4

CHB: chronic hepatitis B infection

**Figure 2:**
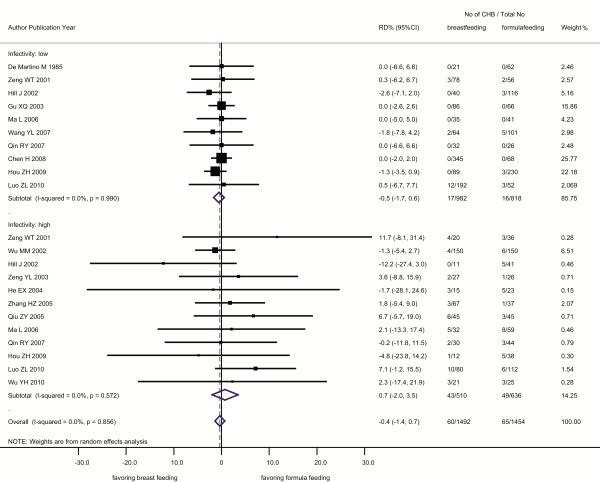
**Effect of breastfeeding on occurrence of chronic hepatitis B (CHB) infection among the infants born of CHB mothers with high and low HBV infectivity analyzed by a random-effects model**. RD: risk difference; CI: confidence interval; CHB: chronic hepatitis B.

Data on HBV infectivity available from 16 of the 32 studies showed that 42.0% (1648/3924) of mothers were designated with "high infectivity", being positive for either the HBeAg and/or the HBV DNA, and that the proportion of these mothers was lower among those who elected to BF (36.9%, 739/2001) than those who elected to FF (44.1%, 812/1843) (Table 2). The overall RD was -0.4% (95% CI: -1.4%, 0.7%), which was not modified by language of paper, quality and type of study, study sites or infant-mother vaccination by both subgroup analysis and Meta regression. And the RD determined for mothers with high infectivity was 0.7% (95% CI: -2.0%, 3.5%), which was not significantly different (Z = 0.789, *P *> 0.05) from that determined for mothers with low infectivity, -0.5% (95% CI: -1.7%, 0.6%) (Figure 2).

### 4. Publication bias and its correction

**Figure 3:**
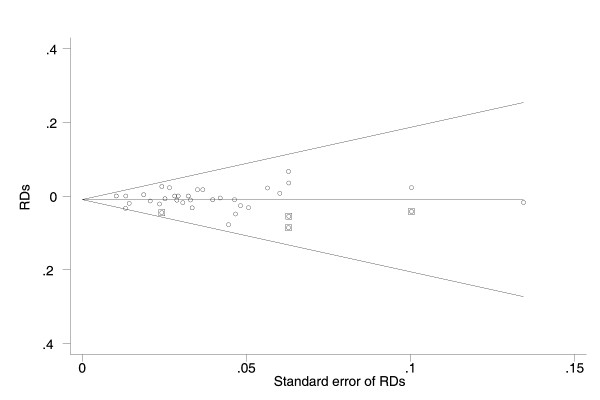
**The Egger's funnel plot with pseudo 95% confidence limits for the 32 studies on the effect of breastfeeding on occurrence of chronic hepatitis B (CHB) infection among the infants born of CHB mothers**. RD: risk difference; CI: confidence interval; CHB: chronic hepatitis B. The circles alone and enclosed with square represented respectively the included and filled studies.

**Figure 4:**
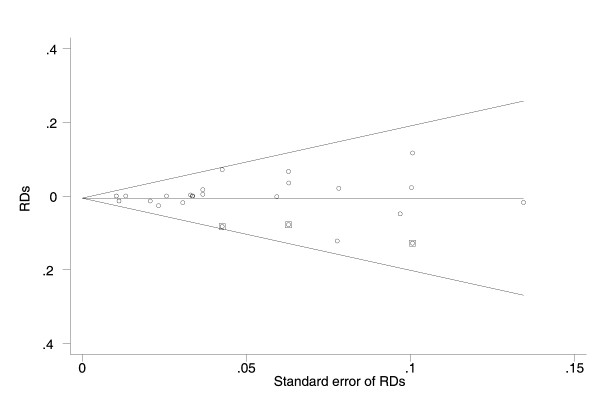
**The Egger's funnel plot with pseudo 95% confidence limits for the 16 studies on the effect of breastfeeding on occurrence of chronic hepatitis B (CHB) infection among the infants born of CHB mothers with high and low infectivity**. RD: risk difference; CI: confidence interval; CHB: chronic hepatitis B. The circles alone and enclosed with square represented respectively the included and filled studies.

Visual inspection of the funnel plots demonstrated a possible publication bias. After trim and fill methods were performed, the pooled RDs were -1.0% (95%CI: -1.8%, -0.1%) and -0.6% (95%CI: -1.6%, 0.5%) respectively for the 32 and 16 studies (Figure 3, 4).

## Discussion

Our results suggest that breastfeeding by CHB mothers does not pose a significant risk of infection by the virus, provided that the infants have been vaccinated against HBV at birth.

In this meta analysis, the overall transmission rate of HBV from CHB mothers to their infants who had completed routine hepatitis B vaccination was 4.32%, which was similar to those previously reported [3]. Mother-to-infant transmission of HBV can occur prenatally, at birth and postnatally [49], but most commonly infection occurs at the time of delivery. In-utero transmission is very uncommon, except in China and Japan, and often persists after birth, which results in the ineffectiveness of HBV vaccination, i.e., immunoprophylaxis [49,50]. From this perspective, most mother-to-infant transmission occurs before deciding on infant feeding methods. But there is an exception: the neonate can be infected postnatally by household contact with CHB family members [51]. Apparently, breastfeeding would produce more frequent contacts between mother and infant than formula-feeding. Though separation of breastfed transmission of CHB from contact seems to be important in the clarification of the net effect of breastfeeding on infantile CHB infection, this contact transmission occurs only at the successful exchange of body fluid from infected mothers to their infants under some special situations, such as excoriation. Thus breastfed transmission of HBV, if existed, should be minimal.

Two previous meta analyses reported different associations of breastfeeding and infantile CHB infection with an odds ratio (OR) = 1.68 (95%CI: 1.16, 2.44) [52] and 0.67 (95%CI: 0.41, 1.12) [53] respectively based on 13 and 10 studies. The inconsistent results reflected difference between the studies with respect to their study aim, search strategies, criteria of inclusion and exclusion of the studies, and analysis strategies. One study included both vaccinated and not vaccinated infants [52] and the other included only those who had been given both passive and active immunization [53]. After careful evaluation on these two analyses, we identified their obvious shortcomings: (1) many eligible studies were not included in these analyses without any reason; (2) the analyses excluded those studies with zero outcome of infantile CHB infection in both groups of feeding methods; (3) neither of the studies evaluated the quality of the included studies and their influence on the results, and (4) neither of the studies considered the possible confounding of mothers' infectivity, which was important in mother-to-infant transmission of HBV and their decisions on how to feed their infants [17,54]. In our meta analysis, reference search, data extraction and quality evaluation were done by two independent investigators and examined by the third one before a consensus was reached, thus the completeness and appropriateness of the studies included for analysis were ensured. We used RD instead of OR or risk ratio (RR) as effect measure, which ensured that a reasonable estimation of effect measurement remained untouched and the 2^nd ^shortcoming mentioned above was overcome simultaneously.

Our findings have important implications for current clinical practice about breastfeeding by CHB mothers, even with high infectivity. Routine hepatitis B vaccination allows the infant to enjoy breastfeeding, the nutritional, immunological and psychological advantages of which are well known[4]. And breastfeeding would also greatly benefit the mother[4], especially in weakening or eliminating her fear and guilt which occur often. Unfortunately, contrary to our findings that risk of breastfed transmission of infantile CHB infection simply does not exist, the current clinical practices seem discouraging: (1) any breastfeeding rate is about 30% lower for those mothers with than without CHB [54], and even lower (5.4% only) for those with high infectivity [55]; (2) there is still 25%-50% of the medical professionals did not recommend infantile breastfeeding by CHB mothers [15,16]. Thus, health education of breastfeeding with correct knowledge, attitude and behaviour on the decision makers, such as medical professional and CHB mothers, seems to be urgent, especially in those epidemic countries, like China.

This meta analysis demonstrates no association between breastfeeding and risk of infantile CHB infection from a CHB mother to her infant. Two alternative explanations must be considered. First, a systematic bias may be present in follow-up studies arising from differential lost to follow-up, which has not been mentioned in most of the studies. Second, the studied population varies from study to study, and some unknown confounders may not be effectively evaluated. Further evaluation of the effect of breastfeeding from an HBV infected mother and infantile CHB infection while taking into consideration of potential biases can be feasibly achieved with more large-scale prospective follow-up studies.

## Conclusions

Breast milk is infectious; yet, breastfeeding, even by mothers with high infectivity, is not associated with demonstrable risk of infection of infantile CHB. This may be partly because infection mainly occurs during delivery and the protection by vaccination. The finding concurs with that of individual studies and supports the WHO recommendation of BF, irrespective of the HBV status of the mothers.

## Competing interests

The authors declare that they have no competing interests.

## Authors' contributions

YJZ conceived, coordinated the study and drafted the manuscript. YL participated in the design and data analysis. QY and YQZ participated in the design, reference search and data extraction. YX participated in quality rating and data analysis. QQY and SW participated in quality rating, data extraction and analysis. All authors read and approved the final manuscript.

## Pre-publication history

The pre-publication history for this paper can be accessed here:

http://www.biomedcentral.com/1471-2458/11/502/prepub

## Supplementary Material

Additional file 1 **Figure S1 Flow chart for the process of retrieving papers in our meta analysis**. CBM: Chinese BioMedical Literature database, CNKI: Chinese National Knowledge Infrastructure. Click here for file
