# Supplementary figures and images for: Should chronic hepatitis B mothers breastfeed? a meta analysis
Source: BMC Public Health. 2011 Jun 27;11:502. doi: 10.1186/1471-2458-11-502 (PMC3141463; doi:10.1186/1471-2458-11-502)

Additional file 1 – Figure 1. Flow chart for the process of retrieving papers in our meta analysis


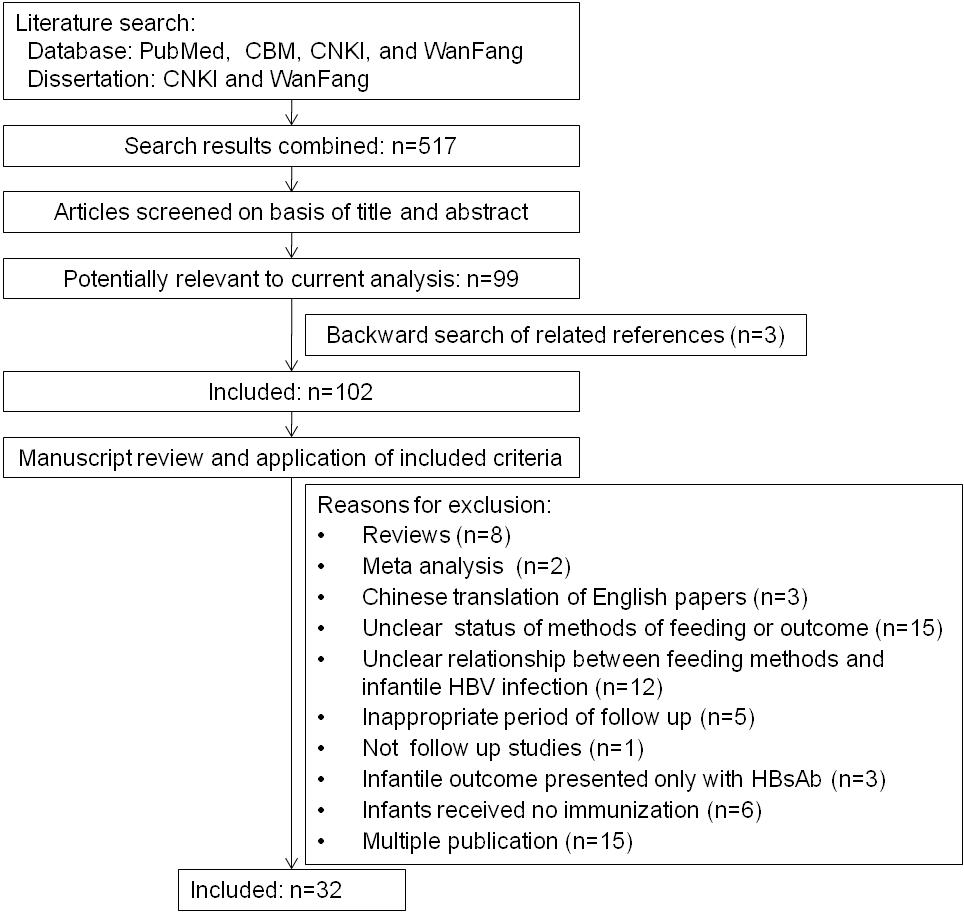

Supplement: Additional file 1 — Figure S1 Flow chart for the process of retrieving papers in our meta analysis. CBM: Chinese BioMedical Literature database, CNKI: Chinese National Knowledge Infrastructure. [file 1471-2458-11-502-S1.DOCX]
